# Study protocol: fish oil supplement in prevention of oxaliplatin-induced peripheral neuropathy in adjuvant colorectal cancer patients – a randomized controlled trial. (OxaNeuro)

**DOI:** 10.1186/s12885-024-11856-z

**Published:** 2024-02-03

**Authors:** Nina Lykkegaard Gehr, Páll Karlsson, Signe Timm, Signe Christensen, Christian Andreas Hvid, Jana Peric, Torben Frøstrup Hansen, Lotte Lauritzen, Nanna Brix Finnerup, Lise Ventzel

**Affiliations:** 1 Danish Pain Research Center, Department of Clinical Medicine, Aarhus University, Aarhus, Denmark; 2 Department of Nutrition, Exercise and Sports, University of Copenhagen, Copenhagen, Denmark; 3 Department of Oncology, Vejle Hospital, University Hospital of Southern Denmark, Vejle, Denmark; 4 Department of Oncology, Aalborg University Hospital, Aalborg, Denmark; 5 Department of Oncology, Aarhus University Hospital, Aarhus, Denmark; 6 Department of Oncology, Soenderborg Hospital, University Hospital of Southern Denmark, Soenderborg, Denmark

**Keywords:** Chemotherapy-induced peripheral neuropathy, Oxaliplatin, Colorectal cancer, Fish oil, Omega-3 fatty acids, Neurofilament light, Inflammation, Specialized proresolving mediators, Quality of life, Randomized controlled trial

## Abstract

**Background:**

Oxaliplatin-induced peripheral neuropathy (OIPN) in general and painful OIPN in particular is a debilitating late effect that severely affects cancer survivors’ quality of life and causes premature cessation of potentially lifesaving treatment. No preventive treatments and no effective treatment for chronic OIPN exist despite many attempts. One of several suggested mechanisms includes neuroinflammation as a contributing factor to OIPN. Fish oil containing long-chain n-3 polyunsaturated fatty acids (n-3 LCPUFAs) are precursors to specialized proresolving mediators that mediate the resolution of inflammation. Our primary hypothesis is that a high supplementation of n-3 LCPUFAs will lower the prevalence and severity of OIPN.

**Methods:**

The OxaNeuro project is an investigator-initiated, multicenter, double-blinded, randomized, placebo-controlled clinical study. We will include 120 patients eligible to receive adjuvant oxaliplatin after colorectal cancer surgery. Patients will receive fish oil capsules containing n-3 LCPUFAs or corn oil daily for 8 months.

The primary endpoint is the prevalence of OIPN at 8 months defined as relevant symptoms, including one of the following: abnormal nerve conduction screening, abnormal vibration threshold test, abnormal skin biopsy, or abnormal pinprick test. Additional endpoints include the intensity and severity of OIPN-related neuropathic pain, patient-reported OIPN symptoms, quality of life, mental health symptoms, body composition, and cognitive evaluation. Furthermore, we will evaluate inflammatory biomarkers in blood samples and skin biopsies, including the potential OIPN biomarker neurofilament light protein (NfL) which will be measured before each cycle of chemotherapy.

**Discussion:**

If readily available fish oil supplementation alleviates OIPN prevalence and severity, it will significantly improve the lives of both cancer survivors and palliative cancer patients receiving oxaliplatin; it will improve their quality of life, optimize chemotherapeutic treatment plans by lowering the need for dose reduction or premature cessation, and potentially increase survival.

**Trial registration:**

ClinicalTrial.gov identifier: NCT05404230

Protocol version: 1.2, April 25^th^. 2023

**Supplementary Information:**

The online version contains supplementary material available at 10.1186/s12885-024-11856-z.

## Background

Symptoms of chemotherapy-induced peripheral neuropathy (CIPN) are generally described as primarily sensory symptoms with a stocking and glove distribution comprising loss of sensation, para- or dysesthesia, and/or pain [1]. CIPN comprises heterogeneous subgroups characterized by varying prevalence and phenotypical manifestations, contingent upon the specific neurotoxic chemotherapy employed [2].

Oxaliplatin-induced peripheral neuropathy (OIPN) is seen in an acute and a chronic version. Acute OIPN occurs in 85-95% of patients receiving oxaliplatin [3]. It is characterized as an acute cold allodynia in the extremities and perioral area. Symptoms occur shortly after treatment and normally dissolve within the following week [4, 5]. The chronic version affects over half of patients receiving oxaliplatin, of whom 20% experience neuropathic pain [6]. Chronic OIPN is a dose- and length-dependent peripheral neuropathy that develops during and often after ended treatment (coasting), where patients report loss of sensation, persistent para- or dysesthesia and/or pain primarily in the feet [6–8]. Cramps and muscle weakness can also be seen in patients [9, 10]. Chronic OIPN, including neuropathic pain, is a disabling condition known to severely affect cancer survivors’ quality of life and is often associated with other problems, such as loss of function, anxiety, depression, and disturbed sleep. Chronic OIPN is associated with a significant human and economic burden on health resources [11, 12].

Despite numerous efforts, no proposed treatments have successfully prevented or effectively managed CIPN [13, 14]. Duloxetine is the only medication that has shown some effect on painful CIPN, but its estimated effect size is low, and the treatment is not without side-effects [15, 16].

Several biomarkers have been assessed in an attempt to find a valid predictor of CIPN [17]. Neurofilament light polypeptide (NfL) is a structural protein shed from neurons upon neuroaxonal damage to the bloodstream. This protein has been proven useful as a biomarker in several neurological diseases, e.g., multiple sclerosis, stroke, and Alzheimer's disease [18]. NfL has been found to correlate with evaluated symptoms of CIPN in smaller, primarily observational, studies including breast cancer or gynecological cancer, with patients receiving taxanes [19–23]. Only one study measured NfL in patients with OIPN symptoms; however, this study measured the level of NfL at fixed time points 3 and 6 months after baseline [24]. To our knowledge, we are the first to examine NfL levels in patients receiving oxaliplatin in a prospective manner before each cycle of oxaliplatin, with a relevant population size and long-term follow-up. This design allows us to evaluate the use of NfL as a predictive biomarker and explore if both acute and chronic CIPN affect the level of NfL.

The mechanism of acute OIPN is altered sodium ion channels which produce peripheral nerve hyperexcitability [25–27]. However, the mechanism of chronic OIPN is not known in detail, albeit several overlapping theories have been proposed. One such dominant theory is that mitochondrial damage is induced by oxidative stress in the dorsal root ganglia (DRG), which is not protected by the blood and brain barrier, causing neuronal degeneration [2]. However, neuroinflammation and modulation of the immune response have been shown to be relevant mechanisms of the pathogenesis of OIPN and the development of neuropathic pain [28]. Several studies have demonstrated an increased innate neuroimmune response and proinflammatory cytokine expression following chemotherapy exposure [29]. In preclinical studies, these changes have been shown to be associated with sensitization and hyperexcitability in peripheral nerves primarily mediated by satellite glial cells surrounding the DRG [30, 31].

Long-chain n-3 polyunsaturated fatty acids (n-3 LCPUFAs), such as docosahexaenoic acid (DHA, 22:6n-3) and eicosapentaenoic acid (EPA, 20:5n-3), have been shown to be potent inflammation inhibitors in animal and cell models [32]. In clinical trials, DHA and EPA have been shown to alleviate chronic inflammatory diseases, such as rheumatic arthritis, by reducing the need for non-steroid inflammatory drugs (NSAIDs) [33]. Additionally, in the acute setting of sepsis, cytokine levels and length of hospital stay were positively affected by DHA and EPA [34].

In cancer patients receiving n-3 LCPUFAs from fish oil supplements, inflammatory cytokines, including interleukin- 6 (IL-6), C-reactive protein (CRP) and tumor necrosis factor-alpha (TNFα), were found to be lower than those in control groups [35]. Eicosanoid-like molecules derived from n-3 LCPUFAs and n-6 LCPUFAs play a crucial role in signaling between immune cells during the inflammatory process. Proinflammatory eicosanoids, such as prostaglandins, thromboxanes and leukotrienes, primarily derived from arachidonic acid (AA, 20:4n-6), are upregulated by early inflammatory stimuli and mediate the initiation of the inflammatory process. The n-3 LCPUFAs, on the other hand, are main precursors for specialized pro-resolving mediators (SPM), resolvins, maresins, and protectins. These SPMs actively mediate downregulation and resolution of the inflammatory process [34]. Hence, an intake of fish oil supplements containing n-3 LCPUFAs increases concentrations of SPMs in peripheral blood and mediates translational changes in immune cells [36]. However, the exact mechanisms of SPMs remain unclear and multifaceted. SPM has also been investigated in the context of neuropathic pain [37]. In this context, a few clinical studies have shown promising results of dietary n-3 LCPUFAs on prevention of CIPN, which should be tested in a larger trial [38–40].

DHA is essential for optimal development and functioning of the nervous system [32, 41], and n-3 LCPUFAs have been shown to affect cognitive development [42]. Some studies have observed beneficial effects on cognitive decline [43], and a meta-analytic review found a significantly positive effect of n-3 LCPUFAs on depression. However, the effect of n-3 PUFAs on cognition during and after chemotherapy has not been evaluated.

N-3 LCPUFAs and chemotherapeutic treatment has been studied with various focus throughout the years. To date no negative interactions between n-3 LCPUFAs and chemotherapy have been reported. Conversely, some preclinical studies indicate a synergistic relationship between the two in certain settings [44, 45].

The primary aim of this study is to evaluate whether a daily intake of a high dosage of fish oil containing n-3 LCPUFAs reduces the prevalence and severity of OIPN and neuropathic pain 8 months after adjuvant oxaliplatin following surgery for high-risk colorectal cancer. Additional aims are to investigate whether n-3 LCPUFAs influence body composition, cognition, and mental status. Furthermore, we want to study the inflammatory mechanism of OIPN in skin biopsies and blood and explore potential early predictive biomarkers of OIPN, particularly NfL.

### Design and methods

Project OxaNeuro is an investigator-initiated, multicenter, double blinded, randomized placebo-controlled clinical trial. Patients are randomized to one of two arms: fish oil capsules or identical corn oil capsules for 8 months in total.

**Fig. 1:**
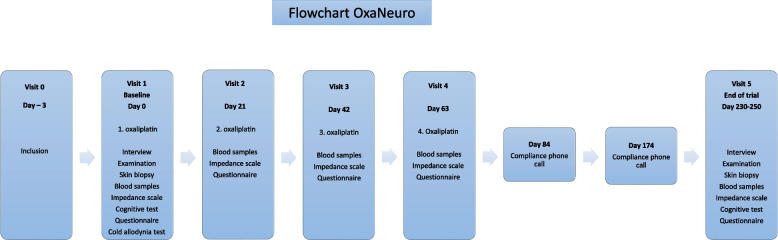
Project OxaNeuro flowchart. Flowchart of the OxaNeuro project. Each blue box represents a patient contact in the project comprising either personal appearance or phone call. One visit (visit 5) is not scheduled together with the planned visits at the Department of Oncology

The study comprises daily intake of capsules, six visits, neurological and sensory examination, questionnaires, skin biopsies and blood sampling. Five of the six visits are scheduled together with planned visits at the Department of Oncology. Adjuvant chemotherapy is concluded after visit four, but the patients will continue with the daily intake of the trial supplements for a total of 8 months. During the follow-up period, patients will receive two phone calls to monitor compliance. The last visit (visit 5) is planned after 8 months, and this concludes the trial period (Fig. 1).

### Study population and eligibility

Patients diagnosed with colorectal cancer, operated, and referred to one of four participating Departments of Oncology at University Hospital of Southern Denmark Vejle, University Hospital of Southern Denmark Soenderborg, Aarhus University Hospital, and Aalborg University Hospital are eligible. Patients meeting the inclusion criteria (Table 1) will be invited to participate and provide written informed consent.

**Table 1:** Inclusion and exclusion criteria

Inclusion criteria	Exclusion criteria
• Histopathologically verified adenocarcinoma of the colon or rectum and planned standard adjuvant treatment with capecitabine in combination with oxaliplatin • Age ≥ 18 • ECOG performance status 0-2 (measurement of a patient’s function in terms of self-care, daily activity and physical ability) [46] • Written and orally informed consent.	• Inability to speak, read, and understand Danish • Previous treatment with neurotoxic chemotherapy • Neurological (including neuropathy) or psychiatric disorders, diabetes, or other significant medical conditions • Alcohol or drug abuse • Sensory disturbances in the feet • Spinal stenosis • Vascular disease (Fontaine grade II or more) • Known allergy to fish, fish oil or corn oil • Fertile patients not willing to use effective methods of contraception during treatment or abstinence • Daily intake of oil supplements and not willing to stop during the trial period • Lack of consent to skin biopsy

ECOG: Eastern Cooperative Oncology Group

Dietary intake of LCPUFAs in the Danish population is low and thus not expected to affect the trial. Therefore, dietary intake is not documented, and specific diets are not cause for exclusion [47, 48].

If patients experience verified disease relapse, they will be withdrawn from the trial. Dose reduction or premature cessation of oxaliplatin is not cause for exclusion if the patient has received at least one dose of oxaliplatin.

### Randomization and blinding

Randomization is carried out automatically 1:1 and stratified to the study site via the Research Electronic Data Capture (REDcap) randomization module and is fully blinded (patient, health care provider, investigator). In case of a medical emergency and proper treatment requires knowledge of the treatment randomization, unblinding is possible via the hospital pharmacy which has randomization envelopes. At the end of the trial period and subsequent data analysis, unblinding will take place.

### Intervention

Intervention capsules consist of fish oil containing 500 mg EPA, 20:5n-3 and 250 mg DHA, 22:6n-3 each. A daily dose of four capsules will supply 2 g EPA and 1 g DHA. The control capsules consist of 1 g corn oil of which 52% is PUFA n-6. The daily control dose of four capsule is 2.08 g n-6 PUFA (linoleic acid) The corn oil dosage is equivalent to normal dietary intake and has no known effect on the examined parameters.

Intake of active or control capsules continues until 8 months after enrollment. No titration or tapering off is needed.

Active and control capsules are manufactured by EPAX Norway a/s (Aalesund, Norway). Monitoring of durability and verification of content in supplied capsules will be carried out by the supplying company EPAX. Capsules are labeled by the hospital pharmacy Vejle Hospital, according to Eudralex - Volume 4, Annex 13.

### Compliance

Compliance will be monitored continuously through verification and registration by a study nurse at each visit. The excess capsules will be counted, and patients will be asked about their compliance at each visit. During the trial two phone calls will be placed with the purpose of compliance and patient retention.

Furthermore, in a subgroup, we will evaluate compliance in blood samples taken at three different time points (visits 1, 4 and 5, see Fig. 1). The n-3 LCPUFA content in erythrocyte membrane is measured as an objective measure of compliance. The samples will be analyzed by high-throughput fatty acid analysis by gas chromatography with flame ionization detection using Varian 3900 or Agilent 7890A instruments [49]. Samples are stored and shipped in bulk for analysis at the Laboratory of Nutritional Lipidomics, Department of Kinesiology and Health Sciences, Faculty of Health, University of Waterloo, Canada.

### Assessment plan

The study procedures are summarized in Fig. 1.

Baseline assessments include patient history and current symptoms, neurological examination including evaluation of cold allodynia, muscle strength in legs, pin prick testing, vibration detection threshold testing, and DPN check equivalent to nerve conduction testing. Furthermore, skin biopsies, cognitive tests and a test for cold allodynia will be carried out. The visit 5 assessment includes the abovementioned tests except the cold allodynia test. At each visit, blood samples and bioimpedance measures will be taken, and patients will be asked to fill out questionnaires.

### Assessment of endpoints

For an overview of endpoints see Supplementary Table 1, Additional file 1.

### Primary endpoint

#### Prevalence of OIPN at 8 months

The primary endpoint is presence of OIPN 8 months after first the oxaliplatin treatment. OIPN is defined as relevant symptoms of OIPN evaluated by a medical professional, plus minimum one of the following: abnormal vibration test, abnormal nerve conduction test by DPN check device, abnormal pinprick test or, abnormal skin biopsy.

*Symptoms of OIPN* patients are interviewed by a medical professional regarding sensory and motor symptoms of polyneuropathy and pain. The predominant symptoms are tingling or pins and needles, numbness, a greater than normal sense of touch, and burning pain with or without cold stimulus.

*Vibration detection threshold (VDT)* evaluation is performed with a Rydel–Seiffer graded tuning fork (64 Hz, 8/8 scale) that is placed on the medial malleolus and left there until the patient is not able to feel a vibrating sensation. The VDT is determined as a disappearance threshold with three stimulus repetitions. The results are compared to a normal material adjusted for age and sex [50].

*Nerve conduction test* is carried out by a point-of-care device, DPN check (NeuroMetric Inc., Woburn, MA), especially developed for sural nerve conduction studies. DPN Check has been validated as a screening tool for the detection of peripheral neuropathy [51].

The device is placed on the skin posterior to the lateral malleolus over an area corresponding to the anatomical distribution of the sural nerve. DPN Check measures conduction velocity (CV) and amplitude of the sensory nerve action potential (SNAP). A response is recorded and defined as abnormal, if the amplitude of the SNAP is measured at less than 1,5 µV or is undetectable.

*The test for pin prick* is carried out by using a Semmes-Weinstein monofilament no 5.88 (bending force 75.9 g/745 mN), (Stoelting, Wood Dale, IL, USA). Patients are asked if the stimulus is either similar to the control stimulus or less or more intense. An abnormal test is defined as any changed sensibility to pin prick compared to baseline [52].

*Skin biopsies* a total of four skin biopsies from the distal leg (10 cm above the ankle) will be obtained from each study participant: two at baseline and two at visit 5. The biopsies will be taken using a disposable 4 mm punch under sterile conditions and upon subcutaneous lidocaine anesthesia. One biopsy from each visit will be used to quantify intraepidermal nerve fiber (IENF) density using the free-floating protocol and PGP 9.5 primary antibody following available guidelines [53]. An abnormal biopsy is defined as IENF density below the 0.05 quantile of normal distribution adjusted for age and sex [54]. Only IENF density at month 8 is considered for the definition and OIPN for the primary outcome.

### Secondary endpoints

#### Intensity of OIPN-related neuropathic pain

Intensity of OIPN-related neuropathic pain at visit 5 (average over the past 24 hours on a 0-10 numeric rating scale (NRS)).

#### Severity of patient-reported OIPN at 8 months

Severity of patient-reported OIPN at 8 months (visit 5) calculated as the change in the European Organization for Research and Treatment of Cancer Quality of Life Questionnaire (EORCT QLQ-CIPN 20) score compared to visit 1 (baseline). The EORTC-QLQ-CIPN20 is a self-reported questionnaire in which CIPN-related symptoms and functional limitations are subjectively scored. The questionnaire has been validated by two large international clinical trials [55, 56] and is recommended for evaluating treatments to prevent CIPN [57]. The questionnaire consists of 20 questions divided into three subscales (sensory, motor, and autonomic) and provides a comprehensive picture of the nature, frequency, and severity of CIPN within the last seven days. The scores will be calculated according to the standard operating procedure of the EORTC and rated from 0 to 100. A higher score is equivalent to a worse outcome/ more symptoms. Sensory symptoms will be evaluated separately on a question-by-question basis comparing answers at visit 1 (baseline) and visit 5.

### Tertiary endpoints

#### Change in patient-reported OIPN symptoms

Further endpoints relate to change in patient-reported CIPN symptoms via the EORTC QLQ-CIPN20 questionnaire at each visit during the trial.

#### Acute cold allodynia

The Ventzel cylinder has been shown to correlate well with symptoms and neurophysiological changes during oxaliplatin exposure [27]. Examination for cold allodynia is performed by holding the Ventzel cylinder, a solid metal cylinder (height 10 cm, diameter 5 cm) kept at 5 degrees Celsius, for 10 seconds. The patients evaluate cold-provoked pain on an NRS scale from 0 (no pain) to 10 (worst possible pain). The baseline assessment is carried out at visit 1 and patients carry out the same test seven consecutive days thereafter and report the results in a diary.

#### Quality of life

Quality of life based on the EORTC QLQ 30 questionnaire at each visit compared to baseline. EORTC QLQ-30 is a self-reported questionnaire assessing the health-related quality of life (HRQoL) of cancer patients in clinical trials. The questionnaire is divided into three subscales assessing global health status, functional scales (physical functioning, role functioning, emotional functioning, cognitive functioning, and social functioning) and symptom scales/items (fatigue, nausea and vomiting, pain, dyspnea, insomnia, appetite loss, constipation, diarrhea, and financial difficulties). HRQoL will be evaluated using the QLQ-C30 at baseline and at each planned visit until the end of the study (visits 1, 2, 3, 4 and 5). The scores will be calculated according to the standard operating procedure of the EORTC and rated from 0 to 100.

#### Mental health symptoms

Symptoms of anxiety, sleep problems and depression are all assessed using the Patient-Reported Outcome Measurement Information System (PROMIS) 6a short form at visits 1 and 5. PROMIS includes questions about symptoms experienced during the previous 7 days with a frequency or severity grading of symptoms. The scores are converted into PROMIS T scores, which are standardized relative to an American/US reference population, and categorized according to impairment (normal, mild, moderate, and severe impairment) [58, 59].

#### Biomarker analysis

Blood samples will be taken at baseline, prior to each cycle of chemotherapy and at 8 months (visits 1, 2, 3, 4 and 5). The samples will be analyzed in bulk for relevant inflammatory biomarkers including circulating SPMs and their downstream protein expression, such as resolvines and neuroprotectins. NfL will be measured and correlated with OIPN, and patient-reported outcomes.

The skin biopsies taken at visits 1 and 5 will be used to quantify IENFD following published guidelines [53]. Additionally, other markers that are relevant to neuropathy may be assessed as well.

#### Body composition

Body composition during fish oil/corn oil intake is measured by a bioimpedance scale. A small electrical impulse (approx. 0.8 mA) is released to the skin in contact with the surface of the scale. Different conductivities and resistances in different tissue types make it possible to calculate body mass index, and the percentage of fat and muscle tissue, respectively. The measurement will be carried out at baseline, prior to each chemotherapy session and at 8 months (visits 1, 2, 3, 4 and 5) [60, 61].

#### Cognitive evaluation

Cognitive evaluation will be carried out at baseline and at 8 months (visits 1 and 5). The tests comprise the trail making test A+B and part of the EORTC-QLQC30 questionnaire concerning cognition. In test A the patient must draw lines to connect circled numbers in a numerical sequence (1-2-3, etc.) as rapidly as possible. In test B the patient must draw lines to connect circled numbers and letters in an alternating numeric and alphabetic sequence (1-A-2-B, etc.) as rapidly as possible. Test scores (finishing time) are compared with normative data stratified by age [62].

#### Documentation of cardiac events

To establish whether a cardiac or thromboembolic event has taken place during the trial period, patients are asked at visit 5. The statement is verified by a specific search in the patient's medical record during the trial period. The search words are “cardiac event”, “AMI”, “STEMI”, “thromboembolic VTE”, and “LE”.

#### Assessment of blinding

At the end of the trial (visit 5), the patient and investigator will be asked which treatment they think the patient has received and the reason for this.

#### Assessment of adverse events

Adverse events will be assessed by the EORTC-QLQ 30 at visits 2, 3, and 4 and by open-ended questions at visit 5.

### Biobank

A biobank will be established at the Laboratory Center, Vejle Hospital for translational research.

The remaining material will be stored for 10 years in a biobank for future research and then destroyed. The biobank is approved by The Region of Southern Denmark.

### Statistical analysis and sample size

The present study will compare the treatment outcomes of patients in the two arms.

Based on previous studies we expect an OIPN prevalence of 50% in the control group and estimate a prevalence in the fish oil group to be 23%, which is a clinically meaningful difference [6, 39, 63]. Using 80% power and 5% risk of type I error, the sample size estimate is 49+49 patients for the primary objective. To account for dropouts, the project will include 120 patients, with 60 patients in each group.

Patient demographics and baseline characteristics will be described using descriptive statistics. Continuous variables will be summarized according to their distribution with medians and interquartile range (IQR) or means and standard deviations (sd). Categorical variables will be summarized with frequencies and percentages.

The primary endpoint (OIPN) is a dichotomous endpoint defined by the developed definition of OIPN. The two independent proportions of OIPN in the two randomization arms will be compared using the chi^2^ test and reported as frequencies and percentages with corresponding p-values. P-values <0.05 will be considered statistically significant. All analyses will be performed as intention-to-treat (ITT). In case of cross-over between study arms due to non-compliance, per protocol analyses will be performed as sensitivity analyses to explore the potential bias from the non-compliance. In per-protocol analyses, non-compliance will be defined using an 80% percent cut-off or a significant lack of DHA and EPA rise in compliance blood samples.

The proportion and patterns of missing data will be explored, and in the case of <5% missing data, analyses will be performed as complete case. If the amount of missing data is comprehensive (>5%) or suggestive of biased estimates, appropriate methods of imputation will be applied depending on the patterns of missingness.

A complete statistical analysis plan covering each outcome will be developed together with statistical consultant Signe Timm M.Sc., PhD. Statistical analyses will be performed using STATA version 18 (STATA Corp., Texas, USA) and all steps of data management, coding and analyses will be logged.

### Ethical considerations

The trial does not affect the treatment regimen or follow-up for the patients. Skin biopsies are minimally invasive. It may cause a short lasting discomfort when placing the local anesthetic, and a small risk of wound infection (1:1.000). The risks involved in blood sampling include a small risk of temporary soreness and hematoma at the point of perforation.

Patients will not be financially reimbursed or compensated.

OIPN is a serious complication with no treatment options. Fish oil and corn oil have no known adverse effects. The group of patients receiving fish oil may benefit from reduced neuropathy caused by the standard chemotherapy.

### Trial status

The protocol is approved by the scientific ethics committee in the Southern Region, Denmark (S20220022, May 5^th^, 2022) and is registered in the clinicaltrials.gov database: NCT05404230.

Inclusion began June 1^st^, 2022, in the first out of four planned sites. Sites two and three were opened for inclusion in January 2023 and in May 2023, respectively. The fourth site is planned to open in October 2023. At present, 15 out of 120 patients have been included. Patient inclusion is planned to continue until the end of 2024.

## Discussion

Project OxaNeuro is a strong randomized, double blinded, placebo-controlled study including a solid biobank on this well-defined group of patients for future research. By defining OIPN and OIPN related neuropathic pain by evaluating both large and small nerve fibers and objectively evaluating nerve fiber density together with relevant symptoms we obtain a comprehensive evaluation of OIPN. We adhere to current guidelines for defining neuropathic pain developed by the International Association of the Study of Pain Special Interest Group on Neuropathic Pain (NeuPSIG) and can establish neuropathic pain in these patients with a high level of certainty [64]. Due to a lack of consensus on the definition and evaluation on chemotherapy induced neuropathy as well as the significant overlap in symptoms, our setup is based on the expert consensus on defining and evaluating diabetic neuropathies [65].

N-3 LCPUFAs have been studied for different purposes preclinically and in several groups of cancer patients, including patients receiving concomitant chemotherapy, and there have been no reports of inferior antineoplastic results in the groups receiving n-3 LCPUFAs [45, 66, 67]. Furthermore, a study comprising almost 26,000 patients found no excess risk of bleeding, cardiovascular events, cancer, or other serious adverse events in patients receiving n-3 PUFAs compared to controls [68]. To achieve the anti-inflammatory effect and pain relief as observed in patients with rheumatoid arthritis, the ratio of EPA/DHA must exceed 1.5 [33]. Additionally, Dempsey et al recommend a combined dosage of EPA and DHA of at least 1000-15000 mg/day for a minimum of 12 weeks to measure elevated levels of EPA and DHA in blood samples [69]. The chosen n-3 LCPUFA dosage in the OxaNeuro project of 2 g EPA and 1 g EPA is based on these data, and the dosage is significantly higher than that in comparable studies [38–40]. However, the European Food Safety Authority (EFSA) reports that a combined daily intake of up to 5 g of DHA and EPA is considered safe for adult consumption [70].

Because fish oil is a supplementation readily available to purchase in various convenience stores, we chose to make the evaluation of compliance objective and thereby more reliable by measuring compliance via blood samples. In the field of supplementation studies, this method is regarded as the benchmark for evaluating compliance [71].

Despite our attempt to address study limitations by adhering to the ACTTION guidelines on CIPN trials [57], we acknowledge several limitations. The duration of the trial and the number of invasive prcodures (skin biopsy and blood samples) can be limitations due to the risk of discontinuations and subsequent inadequate power. Furthermore, power calculations were based on primary outcome only, thereby raising concerns of power for the secondary and tertiary outcomes, which consequently will be regarded as exploratory only.

Since OIPN and neuropathic pain are not manageable or preventable, the current situation in oncology departments today is that OIPN and associated neuropathic pain cause dose reduction and premature cessation of potential lifesaving chemotherapy.

In this study we aim both to explore the mechanisms behind OIPN, evaluate different potential predictive biomarkers and we examine a potential preventive treatment with a robust design. The main goal is to increase quality of life in cancer survivors receiving neurotoxic chemotherapy and potentially optimize treatment with oxaliplatin without invalidating side-effects.

### Supplementary Information

**Additional file 1:** **Supplementary Table 1.** Overview of trial outcomes in the OxaNeuro project. **Supplementary Table 2.** Overview of blood samples and preparation. **Supplementary Table 3.** Appendix 3 Overview of skin biopsies. **Supplementary Table 4.** List of trial sites. **Supplementary Table 5.** World Health Organization Trial Registration Data Set.

## Data Availability

Not applicable.

## Abbreviations

CIPN: Chemotherapy-induced peripheral neuropathy
OIPN: Oxaliplatin-induced peripheral neuropathy
NFL: Neurofilament light protein
DRG: Dorsal root ganglion
NSAID: Non-steroid anti-inflammatory drugs
LCPUFA: long chain polyunsaturated fatty acids
DHA: Docosaexaenoic acid
EPA: Eicosapentaenoic acid
CRP: C-reactive protein
IL: interleukin
TNF: Tumor necrosis factor
SPM: Specialized proresolving mediators
CONSORT: The Consolidated Standards of Reporting Trials
ECOG: Eastern Cooperative Oncology Group
REDcap: Research Electronic Data Capture
EORTC: European Organization for Research and Treatment of Cancer
QLQ: Quality of Life Questionnaire
HRQoL: Health Related Quality of Life
PROMIS: Patient-Reported Outcome Measurement Information System
VDT: Vibration detection threshold
CV: Conduction velocity
SNAP: Sensory nerve action potential
IENFD: Intraepidermal nerve fiber density
NRS: Numeric rating scale
OPEN: Open Patient Explorative Network
IASP: International Association of the Study of Pain
NeuPSIG: Neuropathic Pain Special Interest Group
EFSA: European Food Safety Authority
ICMJE: International committee of medical journal editors

## References

[1] Park SB, Goldstein D, Krishnan AV, Lin CS, Friedlander ML, Cassidy J (2013). Chemotherapy-induced peripheral neurotoxicity: a critical analysis. CA Cancer J Clin.

[2] Li T, Mizrahi D, Goldstein D, Kiernan MC, Park SB (2021). Chemotherapy and peripheral neuropathy. Neurol Sci.

[3] Kang L, Tian Y, Xu S, Chen H (2021). Oxaliplatin-induced peripheral neuropathy: clinical features, mechanisms, prevention and treatment. J Neurol.

[4] Ventzel L, Madsen CS, Jensen AB, Jensen AR, Jensen TS, Finnerup NB (2016). Assessment of acute oxaliplatin-induced cold allodynia: a pilot study. Acta Neurol Scand..

[5] Argyriou AA, Cavaletti G, Briani C, Velasco R, Bruna J, Campagnolo M (2013). Clinical pattern and associations of oxaliplatin acute neurotoxicity: a prospective study in 170 patients with colorectal cancer. Cancer.

[6] Bennedsgaard K, Ventzel L, Themistocleous AC, Bennett DL, Jensen AB, Jensen AR, et al. Long-term symptoms of polyneuropathy in breast and colorectal cancer patients treated with and without adjuvant chemotherapy. 2020(April):5114-23. 10.1002/cam4.3129 PMC7367625 32469145

[7] Ventzel L, Jensen AB, Jensen AR, Jensen TS, Finnerup NB (2016). Chemotherapy-induced pain and neuropathy: a prospective study in patients treated with adjuvant oxaliplatin or docetaxel. Pain.

[8] Staff NP, Grisold A, Grisold W, Windebank AJ (2017). Chemotherapy-induced peripheral neuropathy: A current review. Ann Neurol.

[9] Gewandter. Falls and functional impairments in cancer survivors with chemotherapy-induced peripheral neuropathy (CIPN):a University of Rochester CCOP study. Supp Care Cancer. 2014;21(7):2059-66. 10.1007/s00520-013-1766-y PMC3669650 23446880

[10] Beijers A, Mols F, Dercksen W, Driessen C, Vreugdenhil G (2014). Chemotherapy-induced peripheral neuropathy and impact on quality of life 6 months after treatment with chemotherapy. J Community Support Oncol..

[11] Mols F, Beijers T, Vreugdenhil G. Chemotherapy-induced peripheral neuropathy and its association with quality of life : a systematic review. 2014:2261-9. 10.1007/s00520-014-2255-7 24789421

[12] Shah A, Hoffman EM, Mauermann ML, Loprinzi CL, Klein CJ, Staff NP, et al. peripheral neuropathy in a population-based cohort. 2018;89(6):636-41. 10.1136/jnnp-2017-317215 PMC5970026 29439162

[13] Gewandter JS, Dworkin RH, Finnerup NB, Mohile NA (2017). Painful chemotherapy-induced peripheral neuropathy: lack of treatment efficacy or the wrong clinical trial methodology?. Pain.

[14] Michalova Z, Szekiova E, Blasko J, Vanicky I (2023). Prevention and therapy of chemotherapy-induced peripheral neuropathy: a review of recent findings. Neoplasma.

[15] Finnerup NB, Attal N, Haroutounian S, McNicol E, Baron R, Dworkin RH (2015). Pharmacotherapy for neuropathic pain in adults: a systematic review and meta-analysis. Lancet Neurol.

[16] Smith EML, Pang H, Cirrincione C, Fleishman S, Paskett ED, Ahles T, et al. Effect of Duloxetine on Pain, Function, and Quality of Life Among Patients With Chemotherapy-Induced Painful Peripheral Neuropathy. JAMA. 2013;309(13):135–67. 10.1001/jama.2013.2813 PMC3912515 23549581

[17] Velasco R, Alemany M, Villagran M, Argyriou AA. Predictive Biomarkers of Oxaliplatin-Induced Peripheral Neurotoxicity. J Pers Med. 2021;11(7):1–21. 10.3390/jpm11070669 PMC8306803 34357136

[18] Khalil M, Teunissen CE, Otto M, Piehl F, Sormani MP, Gattringer T (2018). Neurofilaments as biomarkers in neurological disorders. Nat Rev Neurol.

[19] Cavaletti G, Pizzamiglio C, Man A, Engber TM, Comi C, Wilbraham D. Studies to Assess the Utility of Serum Neurofilament Light Chain as a Biomarker in Chemotherapy-Induced Peripheral Neuropathy. Cancers (Basel). 2023;15(17):4216–32. 10.3390/cancers15174216 PMC10486738 37686492

[20] Velasco R, Argyriou AA, Marco C, Mariotto S, Stradella A, Hernandez J, et al. Serum neurofilament levels correlate with electrodiagnostic evidence of axonal loss in paclitaxel-induced peripheral neurotoxicity. J Neurol. 2023;270(1):531–7. 10.1007/s00415-022-11377-4 36094631

[21] Karteri S, Bruna J, Argyriou AA, Mariotto S, Velasco R, Alemany M (2022). Prospectively assessing serum neurofilament light chain levels as a biomarker of paclitaxel-induced peripheral neurotoxicity in breast cancer patients. J Peripher Nerv Syst.

[22] Huehnchen P, Schinke C, Bangemann N, Dordevic AD, Kern J, Maierhof SK, et al. Neurofilament proteins as a potential biomarker in chemotherapy-induced polyneuropathy. JCI Insight. 2022;7(6):e154395. 10.1172/jci.insight.154395 PMC8986065 35133982

[23] Kim SH, Kim KH, Hyun JW, Kim JH, Seo SS, Kim HJ (2022). Blood neurofilament light chain as a biomarker for monitoring and predicting paclitaxel-induced peripheral neuropathy in patients with gynecological cancers. Front Oncol.

[24] Kim S-h, Choi MK, Park NY, Hyun J-w, Lee MY. Serum neurofilament light chain levels as a biomarker of neuroaxonal injury and severity of oxaliplatin-induced peripheral neuropathy. Sci Rep. 2020;14;10(1):7995–8004. 10.1038/s41598-020-64511-5 PMC7224372 32409710

[25] Sittl R, Lampert A, Huth T, Schuy ET, Link AS, Fleckenstein J, et al. Anticancer drug oxaliplatin induces acute cooling-aggravated neuropathy via sodium channel subtype Na(V)1.6-resurgent and persistent current. Proc Natl Acad Sci U S A. 2012;109(17):6704-9. 10.1073/pnas.1118058109 PMC3340057 22493249

[26] Bennedsgaard K, Ventzel L, Grafe P, Tigerholm J, Finnerup NB (2020). Cold aggravates abnormal excitability of motor axonsin oxaliplatin-treated patients. Muscle Nerve.

[27] Heide R, Tankisi H (2018). Axonal excitability changes and acute symptoms of oxaliplatin treatment: In vivo evidence for slowed sodium channel inactivation. Clin Neurophysiol.

[28] Argyriou AA, Bruna J, Park SB, Cavaletti G (2020). Expert Review of Neurotherapeutics Emerging pharmacological strategies for the management of chemotherapy-induced peripheral neurotoxicity ( CIPN ), based on novel CIPN mechanisms. Exp Rev Neurother.

[29] Brandolini L, d'Angelo M, Antonosante A, Allegretti M, Cimini A. Chemokine Signaling in Chemotherapy-Induced Neuropathic Pain. Int J Mol Sci. 2019;20(12):2904 –17. 10.3390/ijms20122904 PMC6627296 31197114

[30] Schmitt LI, Leo M, Kutritz A, Kleinschnitz C, Hagenacker T (2020). Activation and functional modulation of satellite glial cells by oxaliplatin lead to hyperexcitability of sensory neurons in vitro. Mol Cell Neurosci.

[31] Leo M, Schmitt LI, Kutritz A, Kleinschnitz C, Hagenacker T (2021). Cisplatin-induced activation and functional modulation of satellite glial cells lead to cytokine-mediated modulation of sensory neuron excitability. Exp Neurol.

[32] Trépanier M-o, Hopperton KE, Orr SK, Bazinet RP. N-3 polyunsaturated fatty acids in animal models with neuroin fl ammation : An update. Eur J Pharmacol. 2016;785:187-206. 10.1016/j.ejphar.2015.05.045 26036964

[33] Senftleber NK, Nielsen SM, Andersen JR, Bliddal H, Tarp S, Lauritzen L, et al. Marine Oil Supplements for Arthritis Pain: A Systematic Review and Meta-Analysis of Randomized Trials. Nutrients. 2017;9(1):42:63. 10.3390/nu9010042 PMC5295086 28067815

[34] Calder PC (2017). Omega-3 fatty acids and inflammatory processes: from molecules to man. Biochem Soc Trans.

[35] Aguiar JD, Silva P, Emilia M, Fabre DS, Linetzky D (2021). Omega-3 supplements for patients in chemotherapy and / or radiotherapy : a systematic review. Clin Nutr.

[36] Souza PR, Marques RM, Gomez EA, Colas RA, De Matteis R, Zak A (2020). Enriched Marine Oil Supplements Increase Peripheral Blood Specialized Pro-Resolving Mediators Concentrations and Reprogram Host Immune Responses: a Randomized Double-Blind Placebo-Controlled Study. Circ Res.

[37] Leuti A, Fava M, Pellegrini N, Maccarrone M (2021). Role of Specialized Pro-Resolving Mediators in Neuropathic Pain. Front Pharmacol.

[38] Esfahani A, Somi M, Ayromlou H, Nikanfar A, Jafarabadi MA. The effect of n-3 polyunsaturated fatty acids on incidence and severity of oxaliplatin induced peripheral neuropathy : a randomized controlled trial. Biomark Res. 2016;4(13):1–9. 10.1186/s40364-016-0066-3 PMC4918070 27340553

[39] Ghoreishi Z, Esfahani A, Djazayeri A, Djalali M, Golestan B, Ayromlou H (2012). Omega-3 fatty acids are protective against paclitaxel-induced peripheral neuropathy : A randomized double-blind placebo controlled trial. BMC Cancer..

[40] Zhang X, Chen H, Lu Y, Xu C, Yao W, Xu L (2020). Prevention of oxaliplatin-related neurotoxicity by omega-3 PUFAs: A double-blind randomized study of patients receiving oxaliplatin combined with capecitabine for colon cancer. Medicine (Baltimore).

[41] Lu D-y, Tsao Y-y, Leung Y-m, Su K-p (2010). Docosahexaenoic Acid Suppresses Neuroinflammatory Responses and Induces Heme Oxygenase-1 Expression in BV-2 Microglia: Implications of Antidepressant Effects for Omega-3 Fatty Acids. Neuropsychopharmacol.

[42] Kim HY, Huang BX, Spector AA. Molecular and Signaling Mechanisms for Docosahexaenoic Acid-Derived Neurodevelopment and Neuroprotection. Int J Mol Sci. 2022;23(9):4635–48. 10.3390/ijms23094635 PMC9100376 35563025

[43] Lauritzen L, Brambilla P, Mazzocchi A, Harslof LB, Ciappolino V, Agostoni C. DHA Effects in Brain Development and Function. Nutrients. 2016;8(1):6–23 10.3390/nu8010006 PMC4728620 26742060

[44] Gurav P, Patade T, Hajare S, Kedar RN (2023). n-3 PUFAs synergistically enhance the efficacy of doxorubicin by inhibiting the proliferation and invasion of breast cancer cells. Med Oncol.

[45] Laviano A, Rianda S, Molfino A, Rossi Fanelli F (2013). Omega-3 fatty acids in cancer. Curr Opin Clin Nutr Metab Care.

[46] Oken M, Creech R, D T. Toxicity and response criteria of the Eastern Cooperative Oncology Group. Am J Clin Oncol. 1982;5(6):649–55 7165009

[47] Norden. Nordic Nutrition Recommendations 2012, Integrating nutrition and physical activity, 5th edition.

[48] Joensen AM, Schmidt EB, Dethlefsen C, Johnsen SP, Tjonneland A, Rasmussen LH (2010). Dietary intake of total marine n-3 polyunsaturated fatty acids, eicosapentaenoic acid, docosahexaenoic acid and docosapentaenoic acid and the risk of acute coronary syndrome - a cohort study. Br J Nutr.

[49] Vuholm S, Teisen MN, Buch NG, Stark KD, Jakobsen J, Molgaard C (2020). Is high oily fish intake achievable and how does it affect nutrient status in 8-9-year-old children?: the FiSK Junior trial. Eur J Nutr.

[50] Rolke R, Baron R, Maier C, Tolle TR, Treede DR, Beyer A (2006). Quantitative sensory testing in the German Research Network on Neuropathic Pain (DFNS): standardized protocol and reference values. Pain.

[51] Kural MA, Andersen ST, Andersen NT, Andersen H, Charles M, Finnerup NB (2019). The utility of a point-of-care sural nerve conduction device for detection of diabetic polyneuropathy: a cross-sectional study. Muscle Nerve.

[52] Gylfadottir SS, Itani M, Kristensen AG, Karlsson P, Kroigard T, Bennett DL (2022). The characteristics of pain and dysesthesia in patients with diabetic polyneuropathy. PLoS One.

[53] Karlsson P. Skin biopsy analysis in diabetic neuropathy. Diabet Neuropathy. 2022;1:79–90.

[54] Lauria G. Intraepidermal nerve fiber density at the distal leg a worldwide normative.pdf>. J Peripheral Nervous Sys. 2010. 10.1111/j.1529-8027.2010.00271.x 21040142

[55] Postma TJ, Aaronson NK, Heimans JJ, Muller MJ, Hildebrand JG, Delattre JY (2005). The development of an EORTC quality of life questionnaire to assess chemotherapy-induced peripheral neuropathy: the QLQ-CIPN20. Eur J Cancer.

[56] Lavoie Smith EM, Barton DL, Qin R, Steen PD, Aaronson NK, Loprinzi CL (2013). Assessing patient-reported peripheral neuropathy: the reliability and validity of the European Organization for Research and Treatment of Cancer QLQ-CIPN20 Questionnaire. Qual Life Res.

[57] Gewandter JS, Brell J, Cavaletti G, Dougherty PM, Evans S, Howie L (2018). Trial designs for chemotherapy-induced peripheral neuropathy prevention: ACTTION recommendations. Neurology.

[58] Pilkonis PA, Choi SW, Reise SP, Stover AM, Riley WT, Cella D (2011). Item banks for measuring emotional distress from the Patient-Reported Outcomes Measurement Information System (PROMIS(R)): depression, anxiety, and anger. Assessment.

[59] HealthMeasures. PROMIS health measures. Availale at: http://www.healthmeasures.net/explore-measurement-systems/promis. Cited 11 Feb 2022.

[60] Kyle UG, Bosaeus I, De Lorenzo AD, Deurenberg P, Elia M, Gomez JM (2004). Bioelectrical impedance analysis–part I: review of principles and methods. Clin Nutr.

[61] Kyle UG, Bosaeus I, De Lorenzo AD, Deurenberg P, Elia M, Manuel Gomez J (2004). Bioelectrical impedance analysis-part II: utilization in clinical practice. Clin Nutr.

[62] Tombaugh T (2004). Trail Making Test A and B: Normative data stratified by age and education. Arch Clin Neuropsychol.

[63] Teng C, Cohen J, Egger S, Blinman PL, Vardy JL (2022). Systematic review of long-term chemotherapy-induced peripheral neuropathy (CIPN) following adjuvant oxaliplatin for colorectal cancer. Supp Care Cancer.

[64] Finnerup NB, Haroutounian S, Kamerman P, Baron R, Bennett DLH, Bouhassira D (2016). Neuropathic pain: an updated grading system for research and clinical practice. Pain.

[65] Tesfaye S, Boulton AJ, Dyck PJ, Freeman R, Horowitz M, Kempler P (2010). Diabetic neuropathies: update on definitions, diagnostic criteria, estimation of severity, and treatments. Diabetes Care.

[66] Fabian CJ, Kimler BF, Hursting SD (2015). Omega-3 fatty acids for breast cancer prevention and survivorship. Breast Cancer Res.

[67] Camargo CQ, Mocellin MC, Brunetta HS, Chagas TR, Fabre MES, Trindade E (2019). Fish oil decreases the severity of treatment-related adverse events in gastrointestinal cancer patients undergoing chemotherapy: a randomized, placebo-controlled, triple-blind clinical trial. Clin Nutr ESPEN.

[68] Manson JE, Cook NR, Lee IM, Christen W, Bassuk SS, Mora S (2019). Marine n-3 Fatty Acids and Prevention of Cardiovascular Disease and Cancer. N Engl J Med.

[69] Dempsey M, Rockwell MS, Wentz LM (2023). The influence of dietary and supplemental omega-3 fatty acids on the omega-3 index: a scoping review. Front Nutr.

[70] Scientific Opinion on the Tolerable Upper Intake Level of eicosapentaenoic acid (EPA), docosahexaenoic acid (DHA) and docosapentaenoic acid (DPA). EFSA J. 2012;10(7):2815–63. 10.2903/j.efsa.2012.2815 PMC13093066 42016086

[71] Gadaria-Rathod N, Dentone PG, Peskin E, Maguire MG, Moser A, Asbell PA (2013). Red blood cell fatty acid analysis for determining compliance with omega3 supplements in dry eye disease trials. J Ocul Pharmacol Ther.

[72] Harris PA, Taylor R, Thielke R, Payne J, Gonzalez N, Conde JG (2009). Research electronic data capture (REDCap)–a metadata-driven methodology and workflow process for providing translational research informatics support. J Biomed Inform.

[73] ICMJE recommendations. Available from: http://www.ICMJE.org/recommendations/.

