# Supplementary material for: Study protocol: fish oil supplement in prevention of oxaliplatin-induced peripheral neuropathy in adjuvant colorectal cancer patients – a randomized controlled trial. (OxaNeuro)
Source: BMC Cancer. 2024 Feb 3;24:168. doi: 10.1186/s12885-024-11856-z (PMC10837958; doi:10.1186/s12885-024-11856-z)
Supplement: Supplementary file 1 — Additional file 1: Supplementary Table 1. Overview of trial outcomes in the OxaNeuro project. Supplementary Table 2. Overview of blood samples and preparation. Supplementary Table 3. Appendix 3 Overview of skin biopsies. Supplementary Table 4. List of trial sites. Supplementary Table 5. World Health Organization Trial Registration Data Set. [file 12885_2024_11856_MOESM1_ESM.docx]

# Supplementary table 1

## Overview of trial outcomes in the OxaNeuro project.

|  | **Outcome** | **Definition** | **Timepoint** |
| --- | --- | --- | --- |
| **Primary outcome** | Presence of OIPN | Present OIPN symptoms plus one of the following:   - abnormal vibration test or, - abnormal nerve conduction test by DPN check device or, - abnormal pinprick test or, - abnormal skin biopsy | Visit 5 |
| **Secondary outcomes** | Intensity of OIPN-related neuropathic pain | Average over the past 24 hours on a 0-10 numeric rating scale (NRS)). | visit 5 |
|  | Severity of patient-reported OIPN | change in the EORCT QLQ-CIPN 20 score compared to visit 1 (baseline) | visit 5 |
| **Tertiary outcomes** | Change in patient reported OIPN symptoms | change in patient reported OIPN symptoms via EORTC QLQ-CIPN20 questionnaire | Visits 1, 2, 3, 4, 5 |
|  | Acute cold allodynia | Using the Ventzel cylinder, patients evaluate cold-provoked pain on an NRS scale from 0 (no pain) to 10 (worst possible pain) every day for 7 days after first chemotherapy treatment | Visit 2 |
|  | Quality of life | Quality of life based on EORTC QLQ 30 questionnaire. | Visits 1, 2, 3, 4, 5 |
|  | Mental health symptoms | Symptoms of anxiety, sleep problems and depression are all assessed using the Patient-Reported Outcome Measurement Information System (PROMIS) 6a short form | Visits 1 and 5 |
|  | Biomarker analysis | Blood samples will be analyzed in bulks for relevant inflammatory biomarkers including circulating SPMs and potential biomarker NfL. | Visits 1, 2, 3, 4, 5 |
|  | Biomarker analysis | Skin biopsies will be used for IENFD quantification along with other relevant markers of neuropathy. | Visits 1 and 5 |
|  | Body composition | measured by a bioimpedance scale | Visits 1, 2, 3, 4, 5 |
|  | Cognitive evaluation | Evaluated by the trail making test A+B and part of the EORTC-QLQC30 questionnaire concerning cognition. | Visits 1 and 5 |
|  | Documentation of cardiac events | Patient statement verified by a specific search in the patient's medical record | Visit 5 |
|  | Assessment of blinding | Patient and investigator are asked which treatment they think the patient has received and the reason for this. | Visit 5 |
|  | Assessment of adverse events | Adverse events are assessed by EORTC-QLQ 30 at visit 2, 3, and 4 and by open-ended questions at visit 5 | Visits 2, 3, 4, 5 |

Overview of primary, secondary, and tertiary outcomes in the OxaNeuro project. OIPN: Oxaliplatin-Induced Peripheral Neuropathy, EORCT QLQ-CIPN 20: European Organization for Research and Treatment of Cancer Quality of Life Questionnaire – Chemotherapy-Induced Peripheral Neuropathy 20, EORTC QLQ 30: European Organization for Research and Treatment of Cancer Quality of Life Questionnaire 30, IENFD: Intra-Epidermal Nerve Fiber Density.

# Supplementary table 2

## Overview of blood samples and preparation

|  | Compliance analysis | Biomarker analysis |  | SPM analysis | |
| --- | --- | --- | --- | --- | --- |
| All visits |  | 2 x 10 mL EDTA tube  2x 10 mL full blood | 1 x 2,7 mL Citrate tube |  | |
| Visit 0, 3 and 4 | 1 x 4 mL Li-heparin tube |  |  | 1x min 10 ml whole blood  In EDTA glass | |
| Preparation | Erytrocyte purification:  Centrifugation at 2300 g for 10 min. at 4^o^ C within 3 hours.  Plasma is transferred to cryo tubes.  Erytrocytes are washed x 3: Add 2-4 ml 150 mM NaCl, 1 mM EDTA, resuspendation, Centrifugation at 2300 g for 5 min. at 4^o^ C., Supernatant plus left-over buffy coat are separated.  1 part 150 mM NaCl, 1 mM EDTA, and 2 drops 0,1% ­­­­­BHT/ethanol per  ml blood and mix  Fill up in cryo tube and NO is added before lid is put on | Plasma:  1^st^ centrifugation 2000g 15 min.  Plasma is separated.  Distribution to cryo tubes.  Serum:  Rest 30 min.  Centrifugation 2000g, 15 min  Distribution to cryo tubes. | 1^st^ centrifugation  2000 g, 15 min.  Within 30 min. at room temp  2^nd^ centrifugation  2500 g 15min.  At room temp.  Platelet poor plasma into 500 µl aliquots | Centrifugation at 2000 g for 15 min at room temp. at 30 min.  Collect plasma. Min 1,5 mL  Distribution in 1 mL aliquots  Snap freeze |  |
|  |  |  |  |  | Collect buffy coat in 1 mL aliquots  Snap freeze |
| Storing | -80^0^ C freezer  Limited durance. Send to analysis every 4 months | -80^0^ C freezer | -80^0^ C freezer | -80^0^ C freezer  Ship with dry ice. | |

Overview of blood samples and preparation in the OxaNeuro project. EDTA: ethylenediamine tetra-acetic acid, NaCl: Natrium Chlorid, BHT: 2,6-Di-tert-butyl-4-methyl-phenol, NO: Nitic Oxide.

# Supplementary table 3

## Appendix 3 Overview of skin biopsies.

|  | IENFD analysis | Other analysis |
| --- | --- | --- |
| Visit 1 | 1 x 4 mm skin biopy | 1 x 4 mm skin biopy |
| Visit 5 | 1 x 4 mm skin biopy | 1 x 4 mm skin biopy |
| Preparation | In Zambonis fixative for 15-24 hours.  Wash in cryoprotectant two times  Stored in cryoprotectant. | Transfer directly to preparation media: Tissue-Tek O.C.T. compound and to dry ice until storage. |
| Storing | 5 ^0^ C refrigerator until analysis. | -80^0^ C freezer |

Overview of the planed skin biopsies and their preparation in the OxaNeuro project

# Supplementary table 4

## List of trial sites

| Site | Site name |
| --- | --- |
| 1 | Department of Oncology, Vejle Hospital, University Hospital of Southern Denmark, Denmark |
| 2 | Department of Oncology, Soenderborg Hospital, University Hospital of Southern Denmark, Denmark. |
| 3 | Department of Oncology, Aarhus University Hospital, Denmark |
| 4 | Department of Oncology, Aalborg University Hospital, Denmark |

Overview of trial sites in the OxaNeuro project.

# Supplementary table 5

## World Health Organization Trial Registration Data Set

| Data category | Information |
| --- | --- |
| Primary registry and trial identifying number | the clinicaltrials.gov database: NCT05404230 |
| Date of registration in primary registry | May 30^th^, 2022 |
| Secondary identifying numbers |  |
| Source(s) of monetary or material support |  |
| Primary sponsor | LV |
| Secondary sponsor(s) |  |
| Contact for public queries | NLG ninalykgehr@clin.au.dk |
| Contact for scientific queries | NLG ninalykgehr@clin.au.dk |
| Public title | Prevention of Oxaliplatin-induced Nerve Damage in the Body's Extremities (OxaNeuro) |
| Scientific title | Fish oil supplement in prevention of oxaliplatin-induced peripheral neuropathy in adjuvant colorectal cancer patients – a randomized controlled trial. (OxaNeuro) |
| Countries of recruitment | Denmark |
| Health condition(s) or problem(s) studied | Oxaliplatin-induced peripheral neuropathy and neuropathic pain |
| Intervention(s) | Fish-oil |
| Key inclusion and exclusion criteria | Inclusion:  Histopathologically verified adenocarcinoma of the colon or rectum and planned standard adjuvant treatment with capecitabine in combination with oxaliplatin, Age ≥ 18, ECOG performance status 0-2 (measurement of a patient’s function in terms of self-care, daily activity and physical ability), Written and orally informed consent.  Exclusion: Inability to speak, read, and understand Danish, Previous treatment with neurotoxic chemotherapy, Neurological (including neuropathy) or psychiatric disorders, diabetes, or other significant medical conditions, Alcohol or drug abuse, Sensory disturbances in the feet, Spinal stenosis, Vascular disease (Fontaine grade II or more), Known allergy to fish, fish oil or corn oil, Fertile patients not willing to use effective methods of contraception during treatment or abstinence, Daily intake of oil supplements and not willing to stop during the trial period, Lack of consent to skin biopsy |
| Study type | Double blinded, randomized controlled study |
| Date of first enrolment | Jun 1 st. 2022 |
| Target sample size | 120 |
| Recruitment status | Recruiting |
| Primary outcome(s) | Present OIPN symptoms plus one of the following:   - abnormal vibration test or, - abnormal nerve conduction test by DPN check device or, - abnormal pinprick test or, - abnormal skin biopsy |
| Key secondary outcomes | Intensity of OIPN-related neuropathic pain  Severity of patient-reported OIPN |
